# Enhanced Hydrogen Generation from Magnesium–Aluminum Scrap Ball Milled with Low Melting Point Solder Alloy

**DOI:** 10.3390/ma16124450

**Published:** 2023-06-18

**Authors:** Olesya A. Buryakovskaya, Grayr N. Ambaryan, Musi Zh. Suleimanov, Alexey B. Tarasenko, Mikhail S. Vlaskin

**Affiliations:** Joint Institute for High Temperatures of the Russian Academy of Sciences, 125412 Moscow, Russia; ambaryan1991@gmail.com (G.N.A.);

**Keywords:** magnesium scrap, low melting point alloy, ball milling, composite materials, microstructure evolution, intermetallic compounds, aqueous salt solutions, hydrogen generation

## Abstract

In this investigation, composite materials were manufactured of mixed scrap of Mg-based alloys and low melting point Sn–Pb eutectic by high energy ball milling, and their hydrogen generation performance was tested in NaCl solution. The effects of the ball milling duration and additive content on their microstructure and reactivity were investigated. Scanning electron microscopy (SEM) analysis indicated notable structural transformations of the particles during ball milling, and X-ray diffraction analysis (XRD) proved the formation of new intermetallic phases Mg_2_Sn and Mg_2_Pb, which were aimed to augment galvanic corrosion of the base metal. The dependency of the material’s reactivity on the activation time and additive content occurred to be non-monotonic. For all tested samples ball milling during the 1 h provided, the highest hydrogen generation rates and yields as compared to 0.5 and 2 h and compositions with 5 wt.% of the Sn–Pb alloy, demonstrated higher reactivity than those with 0, 2.5, and 10 wt.%.

## 1. Introduction

The idea of the global evolution from traditional oil, gas, and coal to ‘zero-carbon’ energy sources has been discussed since the preceding century. Along with that, for the past two decades, no other fuel but hydrogen has aroused such interest and excitement among scientists, public figures, business circles, and citizens following technology trends. Currently, hydrogen is mostly used as a feedstock in chemical and metallurgical industries. However, its importance as an energy carrier is growing year by year. This gas can be used to provide heat and power supply to buildings and propulsion to vehicles. Hydrogen–oxygen combustion results in the formation of water, and not non-condensable gases. It can be derived from hydrocarbons and biomass or obtained from water splitting by electrolysis, photolysis, radiolysis, or thermolysis. That is why the transition to a hydrogen economy is expected to cope with environmental pollution and climate change issues as well as potential fuel supply shortages [1,2,3,4,5,6].

Whilst approaches to hydrogen production and utilization are abundant, modern energy systems still lack efficient methods for storage and transportation of this highly explosive gas that easily escapes many pressurized containing systems [7,8,9]. In recent years, a good deal of various hydrogen storage techniques have been developing. They include the use of geological salt caverns [10], composite high-pressure cylinders [11], cryogenic tanks [12], carbon-based materials [13], metal hydrides [14], metal–organic frameworks [15], hydroreactive ammonia borane and metal–boron hydrides [16], and liquid organic carriers [17]. However, each of the listed methods has a downside. Thus, systems for compressed storage require tremendously high pressure, cryogenic ones demand extremely low temperatures, and other ones have either relatively small capacities or high prices, together with very specific hydrogenation/dehydrogenation conditions [18].

The implementation of hydroreactive metals—primarily, aluminum and magnesium—for hydrogen generation represents quite a different proposition. Along with relatively high hydrogen capacities (11.1 and 8.3 wt.%, respectively), their benefits include abundance and availability, moderate prices, and recovery capability. They are convenient for storing and can be used for the in situ generation of a required hydrogen amount [19,20]. The problem is that, under normal conditions, they barely react with water due to the presence of a protective oxide film on the surface. Another problem is the formation of poorly permeable reaction product depositions onto it. Therefore, to induce the reaction, additional measures are required to remove said barriers [21,22]. One of the said measures involves increasing the reaction temperature over 100 °C in order to enhance water diffusion through the product layer [23,24,25,26]. An alternative widely used approach is based on the chemical dissolution of the product layer in acid, salt or alkali (for aluminum) solutions [27,28,29,30,31,32,33,34]. Another known method is enlarging the specific surface area by application of nanoparticles [35,36] or preparation of fine powders by high-energy ball milling with various ‘assisting agents’ which provide particles’ size reduction and prevent their agglomeration (e.g., NaCl, KCl, BaCl_2_, MgCl_2_, g-C_3_N_4_, graphite, and AlCl_3_) [37,38,39,40,41]. The same principle generally works for aluminum treated with Ga-based alloys which cause its liquid–metal embrittlement (Rehbinder effect) [42,43,44,45], and one more mechanism for the reaction acceleration is to induce galvanic corrosion of aluminum or magnesium in water or aqueous solutions via their coupling with nobler metal additives (Bi, In, Sn, Ni, Ce, La, Cu, etc.) by either alloying or high-energy ball milling [46,47,48,49,50,51,52,53]. 

Along with the transition to cleaner energy carriers, a problem of efficient metal waste recycling exists as well. The majority of the world’s regions are still far from establishing a circular economy ensuring the maximum possible recovery of raw materials or energy from municipal solid waste. Recyclable items such as metals, paper, and plastic can account for as much as 63% of the solid waste stream, and in a number of countries, the current fraction of recycled materials is only ~10% [54,55,56]. Apart from the recovery of valuable resources from disposed of materials, there is another problem associated particularly with aluminum and magnesium waste. Fine dust and micropowders of aluminum, magnesium or some of their alloys are inflammable and explosive. Landfilled aluminum- and magnesium-rich materials coming into contact with landfill leachate may react vigorously to generate pockets of hydrogen that can result in combustion. Therefore, these materials are classified by European regulations as special hazardous wastes and should not be dumped as they are [57,58,59,60,61]. Aluminum dross and scrap can be utilized with hydrogen evolution by its treatment with aqueous alkaline solutions [62,63], while the oxidation of magnesium waste can be performed by saline solutions, natural seawater or a simulated one (3.5 wt.% NaCl solution) [64,65,66]. 

Summarizing the above data, it can be concluded that the chemical utilization of aluminum and magnesium scrap can provide a beneficial process for hazardous waste disposal along with hydrogen generation. In particular, such a process can be used for mixed metal scraps as well as for hardly recyclable alloys with high percentages of alloying elements and low contents of impurities. Magnesium alloy castings are increasingly used in cyclically-loaded structural applications and represent crucial components of automotive, aerospace, and other transportation industries [67,68]. It is noteworthy that about one-third of the magnesium used in the fabrication of structural products ends as scrap, leading to the accumulation of tons of magnesium alloy wastes [69,70]. And those growing amounts of magnesium-based waste have a potential for effective utilization. In the present study, a method for ‘hydrogen recovery’ by the elaboration of hydroreactive samples from mixed scrap of Mg and Mg–Al casting alloys of the ML10 and ML5 grades, generally equivalent to the NZK (Mg–Nd–Zn–Zr) and AZ91D (Mg–Al–Zn), respectively, and their oxidation in aqueous salt solution will be tested. The solution will be represented by 3.5 wt.% NaCl, firstly, because it simulates ecologically friendly seawater, and secondly, because it is a standard media used for many Mg-based materials in the studies on their corrosion behavior (involving hydrogen evolution). Finally, the hydroreactive materials (‘mechanical alloys’) will be prepared by high-energy ball milling using the mentioned mixed scrap as a base material and commercial low melting point Sn–Pb solder alloy ‘POS-61’ as an activating additive. Sn and Pb were reported to enhance hydrogen release accompanying the corrosion of Mg-based alloys due to the formation of intermetallic Mg_2_Sn and Mg_2_Pb phases. With their respective corrosion potentials of −1498 and –1002 mV (measured in 3.5 wt.% NaCl solution), those compounds are ‘cathodic’ to the Mg phase (–1669 mV) [71,72]. Tin and lead are widely available and inexpensive materials. Despite its toxicity, lead is still successfully used in batteries for which special recovery/recycling programs were developed. Some tin compounds can also induce toxic effects; however, roasting, leaching and precipitation procedures have been developed for the effective extraction of those metals [73,74,75]. Low melting point metals (e.g., Bi, Zn, and In) were proved to be good activators for Mg because during ball milling they easily reacted to form complex ‘cathodic’ compounds [76]. The metals under consideration have low melting temperatures (327 °C for Pb, and 232 °C for Sn [77,78]). In the case of solder alloy ‘POS-61’, it is still lower (183 °C for Sn–Pb eutectic [79,80]), which can be beneficial for its effective distribution over the samples’ particles during ball milling. The aim of the research is to investigate the effects of the ball milling duration and additive content on the hydrogen yield and evolution rate of the hydroreactive samples elaborated from waste composed of alloys with rather good corrosion resistance [81,82].

## 2. Materials and Methods

The base material for the hydroreactive samples was composed of shavings and chips of the ML10 and ML5 magnesium alloy grades (National Standard ‘GOST 2856-79’ [83]). The activating additive was a commercial low melting point Sn–Pb solder alloy ‘POS-61′ (National Standard ‘GOST 21930-76’ [84]) with its Sn to Pb mass ratio close to that of the eutectic point. Salt solution (3.5 wt.% NaCl) was prepared of chemically pure NaCl (National Standard ‘GOST 4233-77’ [85], ‘LabTech’ Ltd., Moscow, Russia) and distilled water.

Machine oil was removed from the original scrap with pure acetonitrile (Technical Specification No. 2636-092-44493179-04, ‘EKOS-1’ JSC, Moscow, Russia) under alternating ultrasonic and magnetic mixer agitation. The first step was ultrasonic cleaning (1 h) in an ultrasonic bath sonicator (PSB-2835-05; ‘PSB-Gals’ Ltd., Moscow, Russia). The second step constituted stirring (1 h) via a magnetic mixer (C-MAG; ‘HS 7 IKA-Werke’ GmbH & Co. KG, Staufen, Germany). After that, the used degreaser was removed, a fresh portion was added, and the described steps were performed again. Then, the scrap was separated and dried at room temperature.

The equipment for high energy ball milling comprised a centrifugal ball mill (S 100; ‘Retsch’ GmbH, Haan, Germany), 24 stainless steel 10 mm balls, and a 50 mL grinding jar filled in a glove box (G-BOX-F-290; ‘FUMATECH’ Ltd., Novosibirsk, Russia) under pure argon (99.993%, National State Standard ‘GOST 10157-79’ [86], ‘NII KM’ Ltd., Moscow, Russia). The activation was performed during 0.5, 1, or 2 h under a rotational speed of 580 rpm and a ball-to-powder mass ratio of 24:1.

**Figure 1:**
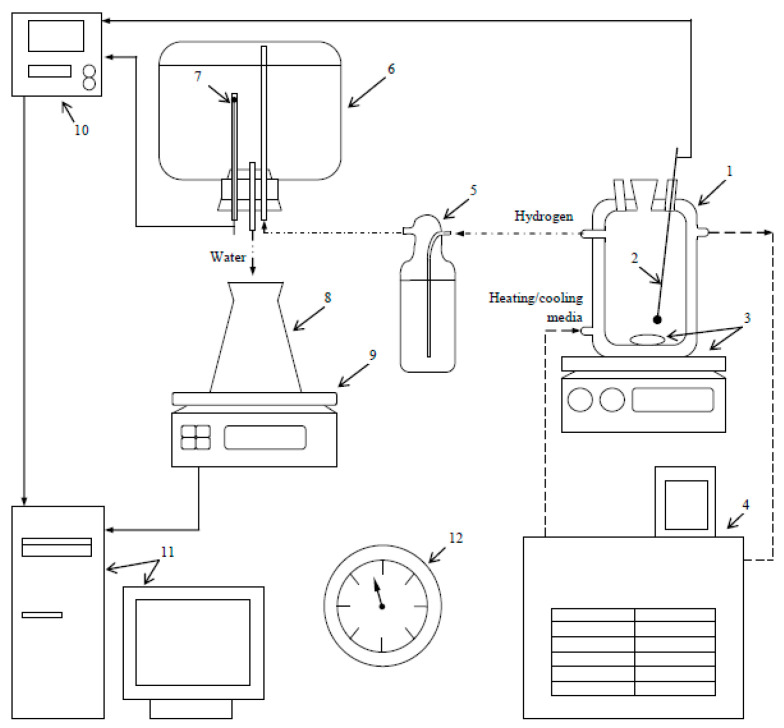
Experimental set: 1—reactor; 2—thermocouple; 3—magnetic mixer and stirring bar; 4—thermostat; 5—Drexel flask; 6—glass vessel; 7—resistance temperature detector; 8—flask; 9—scales; 10—multichannel thermometer; 11—computer; 12—barometer [87].

The experimental set was the same as it was in a preceding study [87] but with a larger glass reactor (1000 mL, JSC ‘Lenz Laborglas’, Wertheim, Germany). The arrangement of the equipment is illustrated in Figure 1. The temperature (25 °C) in the reactor was maintained with a thermostat (CC-308B; JSC ‘ONE Peter Huber Kältemaschinenbau’, Offenburg, Germany), and agitation was provided via a magnetic mixer (C-MAG HS 7; JSC ‘IKA-Werke’, Staufen, Germany). In each experiment, a 0.75 g hydroreactive sample portion was loaded into the reactor. The emerging hydrogen passed through a Drexel flask into a glass vessel from which water was discharged and collected in a flask, of which the mass was continuously measured with scales (ATL-8200d1-I; ‘Acculab Sartorius Group’, New York, NY, USA). A thermocouple (TP.KhK(L)-K11; ‘Relsib’ LLC, Novosibirsk, Russia) and a resistance temperature detector (TS-1288 F/11; ‘Elemer’ LLC, Podolsk, Russia) connected to a multichannel thermometer (TM 5103; ‘Elemer’ LLC, Podolsk, Russia) provided temperature indications. The measured data were transmitted to a computer. The atmosphere pressure was checked using a barometer (BTKSN-18; Technical Specification No. 1-099-20-85, ‘UTYOS’ JSC, Ulyanovsk, Russia). The hydrogen volume at standard conditions (Standard DIN 1343 [88]: 101,325 Pa, 0 °C) was calculated using the ideal gas law. For each sort of sample, three repetitive experiments were carried out to obtain average kinetic curves and standard deviations.

For the samples ball milled without additives, the particle sizes were estimated previously [89] using an optical microscope (Bio 6) with a camera (UCMOS 10,000 KPA; ‘Altami’ LLC, Saint Petersburg, Russia) and ‘Altami Studio 3.5’ software. The procedure included taking images under brightfield illumination, object (particles) contouring, and calculation of the contour sizes (maximum Feret diameters) using the calibration data. The respective specific surface areas were also obtained earlier [89] via a surface area and pore size analyzer Nova 1200e (‘Quantachrome Instruments’ LLC, Boynton Beach, FL, USA). The measured data on low-temperature nitrogen sorption were processed by the ‘Quantachrome NovaWin’ software (version 10.0) employing the Brunauer–Emmett–Teller (BET) equation. Microstructure investigations were performed by scanning electron microscopy (SEM) method under secondary electron (SE) and backscattered electron (BSE) modes (operating voltage 20.0 keV). The elemental compositions were estimated by the energy-dispersive X-ray spectroscopy (EDX) method. The SEM-EDX analysis was carried out by a scanning electron microscope TESCAN VEGA3 (‘Oxford Instruments’ PLC, Abingdon, United Kingdom) for all samples except those ball milled without additives whose images were taken previously by a microscope NOVA NanoSem 650 (FEI Co., Hillsboro, OR, USA). The X-ray diffraction (XRD) analysis was performed at room temperature by a ‘Difraey 401′ diffractometer (‘Scientific Instruments’ JSC, Saint Petersburg, Russia) using Cr-Kα radiation source (0.22909 nm) with the Bragg–Brentano focusing geometry. The scanning was performed in the 2θ angular range from 14 to 140° with a step size of 0.01°. The XRD peaks were identified using a database (Powder Diffraction File™) from the International Centre for Diffraction Data (ICDD).

## 3. Results and Discussion

### 3.1. Properties of the Original Materials and Hydroreactive Samples

In the preceding studies [87,89], it was established that under the same ball milling conditions, prolonged ball milling (2–4 h), together with a relatively high content of additive (20 wt.% of low melting point Wood’s alloy), occurred and had a negative effect on the hydrogen production performance of the samples manufactured from the same magnesium alloy scrap. According to the results represented in [89], the maximum specific surface area corresponded to the original Mg scrap (2.567 m^2^/g). For the samples ball milled for 0.5 and 1 h, it decreased, respectively, to 2.036 and 2.022 m^2^/g. For 2 h of activation a drastic decrease in the specific surface area (<0.1 m^2^/g) was registered, and after another 2 h of activation (4 h in total) that value increased to 0.943 m^2^/g. Such a non-monotonic dependence resulted from the microstructure evolution of the sample particles exposed to the mechanical impacts of milling balls which caused competing processes of agglomeration and disintegration. After 0.5 and 1 h activation the particles represented flattened ‘flakes’ with their average sizes (maximum Feret diameters) of 277 and 155 μm. Those milled for 2 and 4 h represented compacted solid objects with irregular shapes, and the former were significantly larger than the latter ones: 68 μm vs. 36 μm. Taking into account the data set forth above, in the present study the milling duration was limited by 2 h, and the maximum tested additive content was 10 wt.%.

#### 3.1.1. Microstructure Analysis

**Figure 2:**
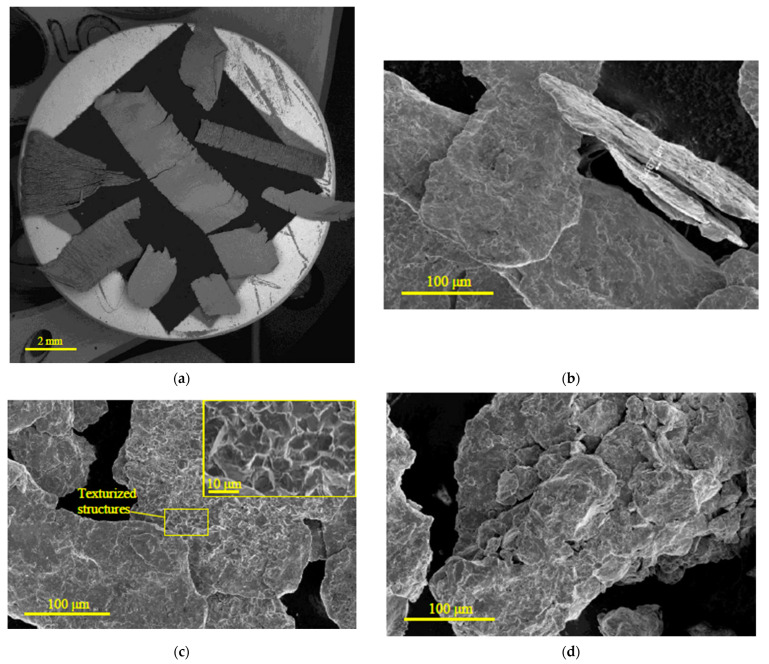

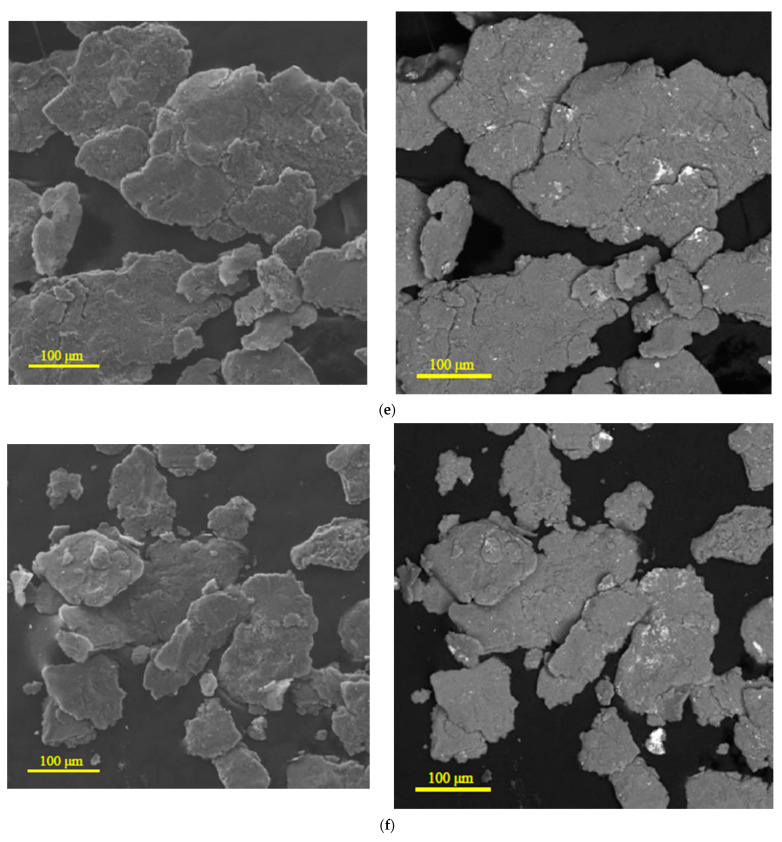

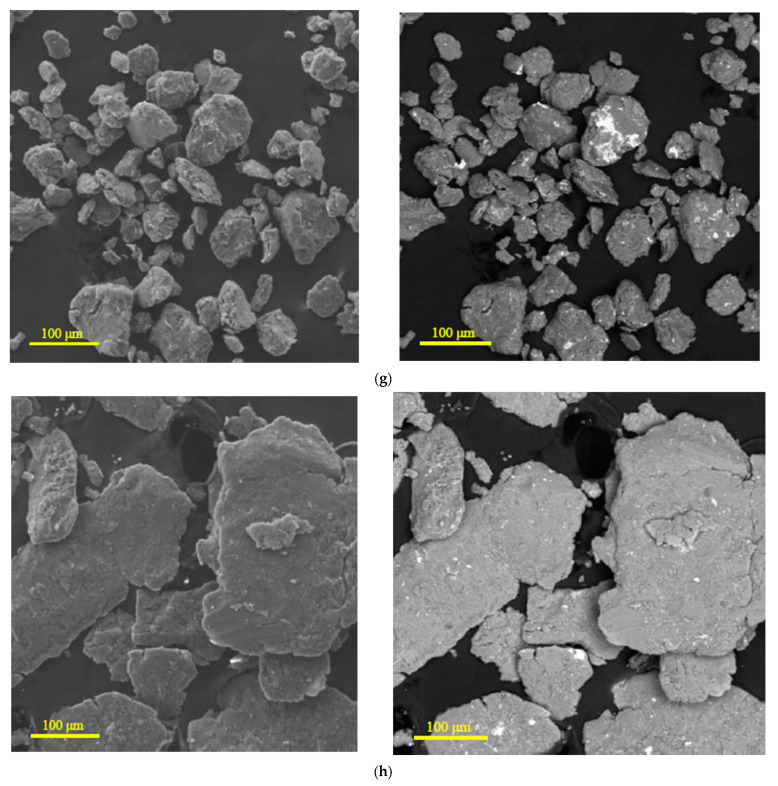

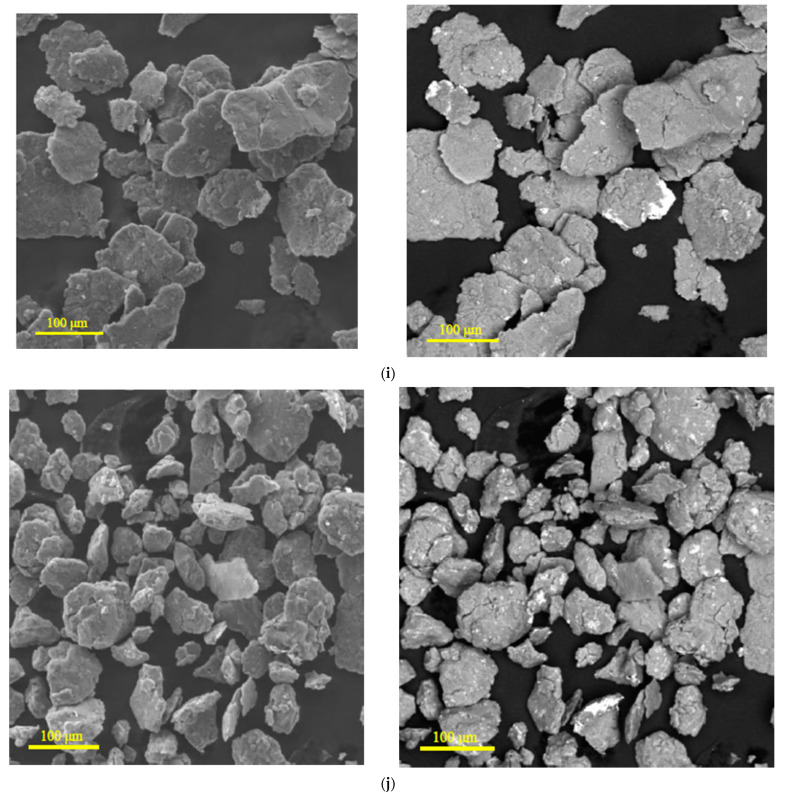

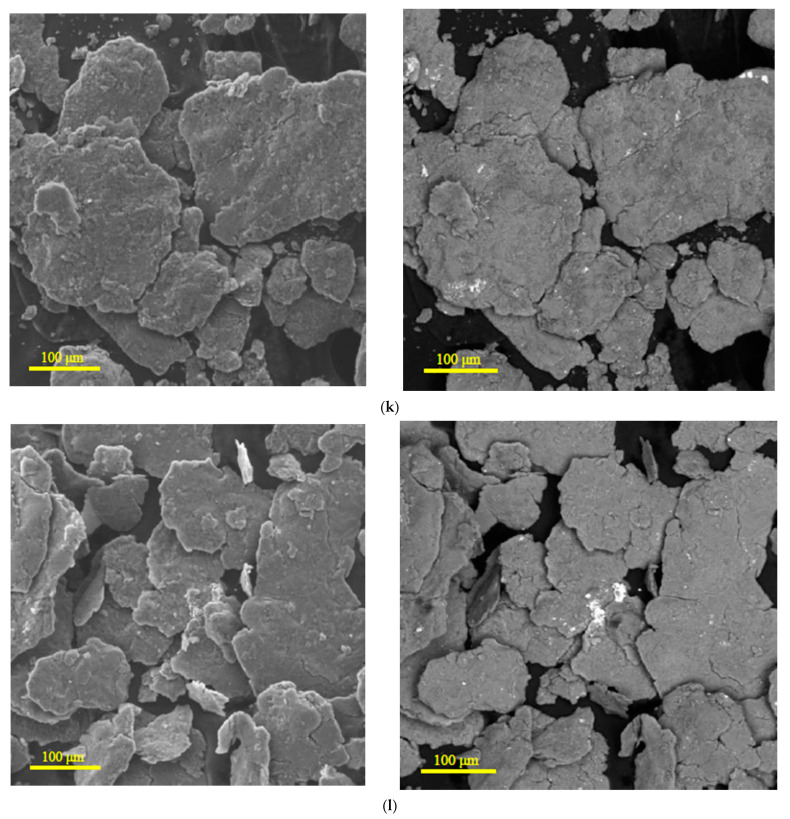

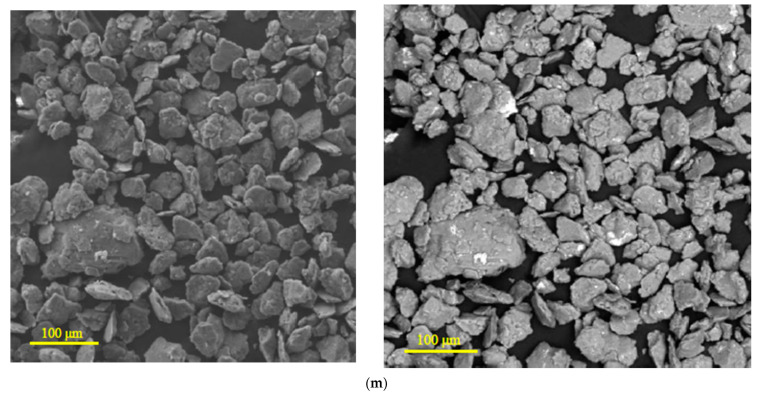
Microhpotorgaphs of the original scrap and ball milled samples: (**a**) original scrap pieces (BSE); (**b**) 0.5 h milled sample without additives (SE); (**c**) 1 h milled sample without additives (SE); (**d**) 2 h milled sample without additives (SE); (**e**) 0.5 h milled sample with 10 wt.% ‘POS-61’ (SE and BSE); (**f**) 1 h milled sample with 10 wt.% ‘POS-61’ (SE and BSE); (**g**) 2 h milled sample with 10 wt.% ‘POS-61’ (SE and BSE); (**h**) 0.5 h milled sample with 5 wt.% ‘POS-61’ (SE and BSE); (**i**) 1 h milled sample with 5 wt.% ‘POS-61’ (SE and BSE); (**j**) 2 h milled sample with 10 wt.% ‘POS-61’ (SE and BSE); (**k**) 0.5 h milled sample with 2.5 wt.% ‘POS-61’ (SE and BSE); (**l**) 1 h milled sample with 2.5 wt.% ‘POS-61’ (SE and BSE); (**m**) 2 h milled sample with 2.5 wt.% ‘POS-61’ (SE and BSE).

The microphotographs of the original scrap and the samples ball milled for 0.5, 1, and 2 h without additives and with different amounts of ‘POS-61’ alloy (10, 5, and 2.5 wt.%) are represented in Figure 2. In the scrap microphotograph, several large particles are shown; however, there were plenty of smaller ones in the starting material portions for ball milling. As can be seen, the original scrap pieces had a lot of traces from mechanical machinings, such as cracks, rough edges, bends, grooves, and scratches. As can be seen in the SE images of the samples milled without additives, after 0.5 and 1 h of activation the particles represented flattened shapes with the surface imperfections caused by steel balls impacts. For the 1 h milled sample, about a quarter of all particles had also texturized (oriented) structures on their surface (see Figure 2c), which presumably resulted from polygonization and recrystallization processes induced by plastic deformation of metal [90,91]. The particles of the 2 h activated sample represented compacted solid shapes formed by the agglomeration of smaller particles generated by the disintegration of the mentioned flattened shapes. The disintegration was caused by fracturing of the particles embrittled by severe plastic deformation, and agglomeration resulted from ‘cold welding’ under the impacts of steel balls. In more detail, the known processes occurring during high-energy ball milling are particles flattening (if they are not originally flat), their agglomeration into lamellar structures, hardening and embrittlement due to the accumulation of plastic deformations, fracturing into smaller pieces, agglomeration of the said smaller pieces by ‘cold welding’, and formation of compacted equiaxed solid objects (which again can be cracked into smaller particles). The final particle size and shape are determined by the equilibrium between cold welding and fracturing [92,93]. 

The pictures of the samples ball milled with the additive (10, 5, and 2.5 wt.% ‘POS-61’ alloy) were taken in the BSE mode (right positions in Figure 2) as well, to demonstrate the presence of the activator. In these images, dark and light grey shades corresponded to the original scrap (mainly composed of Mg and Al), and white spots majorly depicted the heavy activator components, Sn and Pb. Some of the white regions could, however, correspond to contaminating impurities (e.g., Fe) known to be introduced during ball milling with steel balls [94] and heavy alloying elements; for example, Nd-rich precipitates along the grain boundaries, Zr-rich regions, segregated Zn, etc. [95,96]. Originally, the activating additive (‘POS-61’ alloy) loaded into the grinding jar presented in the form of several alloy pieces nearly several mm in size. Considering the alloy’s ductility and relatively low melting point, its distribution among particles was likely majorly attributed to its melting by the heat released during high-energy ball milling rather than to its mechanical disintegration. As to the structural transformations of the particles, the tendency was similar to that for the samples milled without additives, and, for the same milling durations, no notable differences in the particle sizes were observed for the ‘alloyed’ samples and those of scrap only.

#### 3.1.2. Chemical Characterization

**Figure A1:**
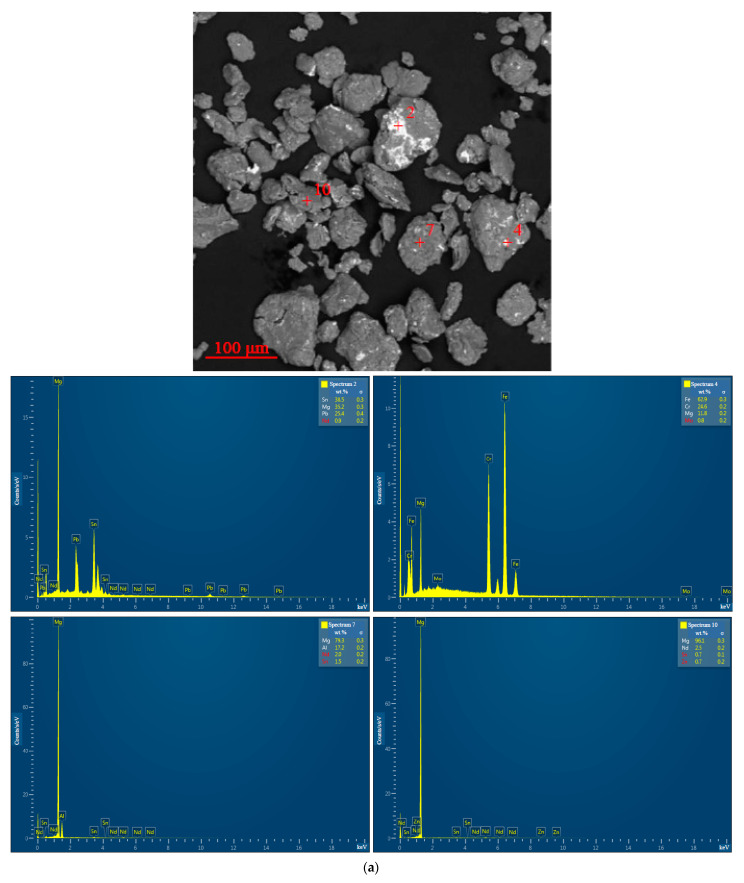

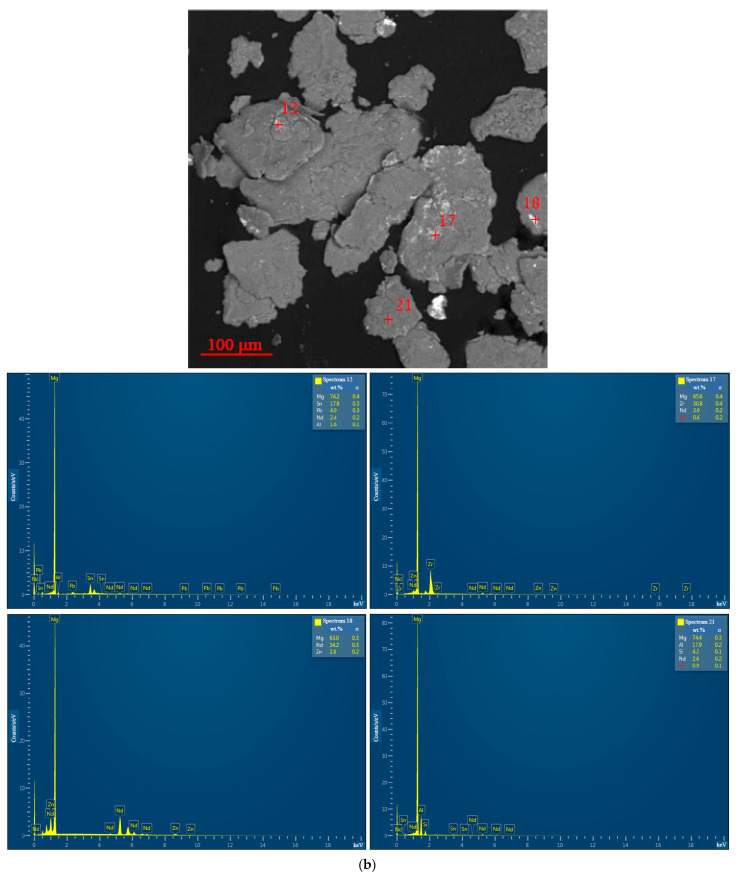

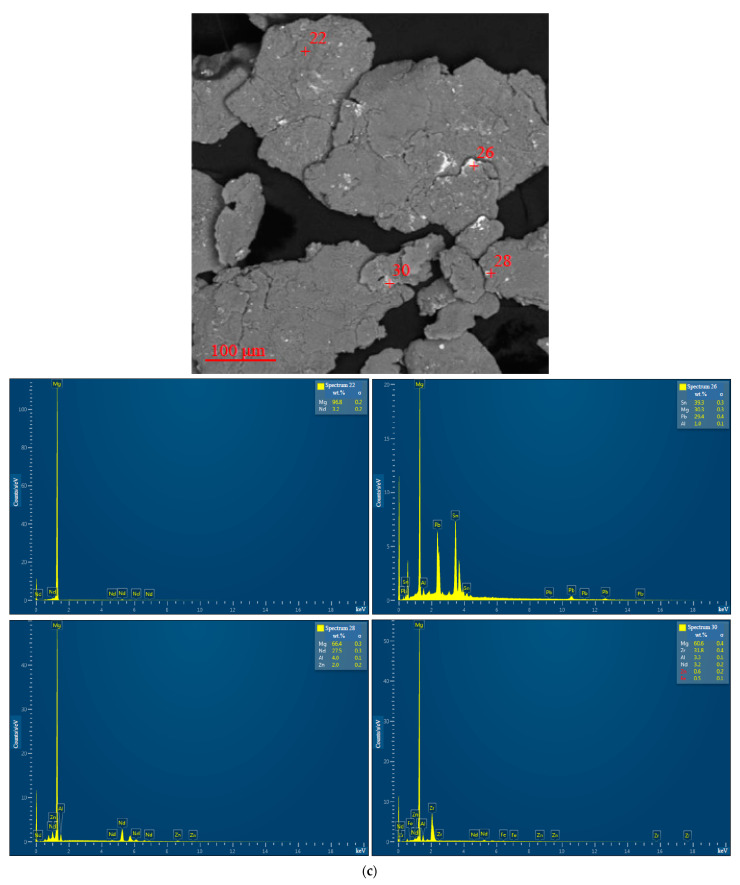
EDX analysis results (spectra) for the compositions with 10 wt.% ‘POS-61’ additive: (**a**) 2 h milled sample; (**b**) 1 h milled sample; (**c**) 0.5 h milled sample.

For the samples ball milled with the ‘POS-61’ alloy, qualitative elemental analysis was performed by the EDX method. For each sample, 10–11 points were inspected. According to the results, the major elements included Mg, Al, Zn, Nd, Zr, Pb, and Sn—main components of the scrap’s Mg-Al-Zn and Mg-Nd-Zn-Zr alloys and Sn-Pb additive. The minor ones included Fe, Cr, Mo, Si, Cu, and Sb. Fe, Si, and Cu were typical impurities of the scrap alloys, and Sb was a standard impurity of the ‘POS-61’ additive. The considerable amount of Fe along with Cr and Mo detected in the samples was likely caused by their contamination with the finest steel particles chipped off from the balls during high-energy ball milling. According to the estimations based on the difference in the ball weights prior to and after milling cycles, the maximum mass fraction for contamination in the samples achieved ~0.2 wt.%. Some of the EDX analysis results for the samples ball milled with 10 wt.% ‘POS-61’ during 2, 1, and 0.5 h are illustrated in Figure A1 (see Appendix A). The spectra for the selected analyzed regions for the samples with 10 wt.% ‘POS-61’ (2, 1, and 0.5 h ball milling) and those with 5 wt.% ‘POS-61’ milled for 2 and 1 h (not shown in Figure A1) are given in Table 1. Some of the tested spots occurred to be highly enriched with the alloying components of the original materials or with impurities introduced by ball milling. The results demonstrated a rather significant non-uniformity in the distribution of the additive components, contaminating impurities, and alloying elements, as was expected for the relatively short milling durations tested in the present study.

#### 3.1.3. Phase Composition

**Figure 3:**
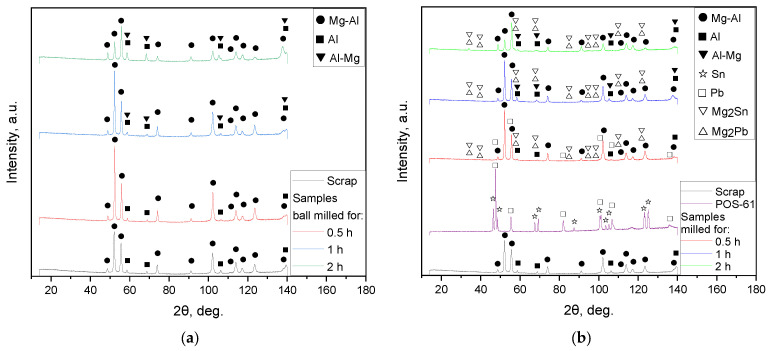

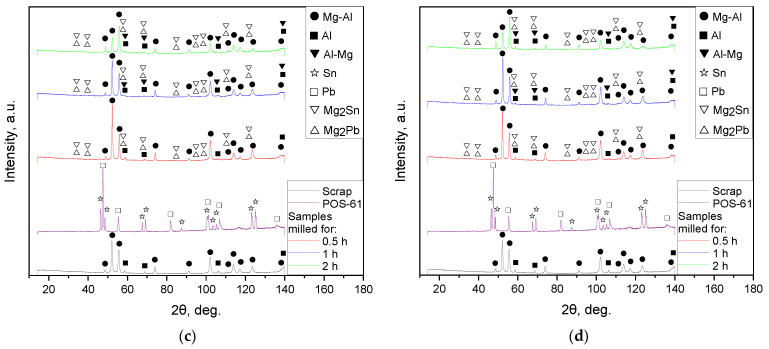
XRD patterns for the original and resulting materials: (**a**) original scrap and samples milled without additive; (**b**) original scrap, ‘POS-61’, and samples milled with 10 wt.% additive; (**c**) original scrap, ‘POS-61’, and samples milled with 5 wt.% additive; (**d**) original scrap, ‘POS-61’, and samples milled with 2.5 wt.% additive.

The XRD patterns for the samples ball milled with no additives as well as for those with 10, 5, and 2.5 wt.% of the ‘POS-61’ alloy are represented in Figure 3. The predominant phase of the scrap and all samples was a solid solution of Al in the primary hexagonal close-packed Mg lattice. Peaks corresponding to the face-centered cubic Al structure were identified as well. Several of the Mg-Al and Al peaks for the scrap, 0.5 and 1 h milled samples had higher intensities than those for the 2 h activated sample, and their relative peak intensities diverged from the ICDD card patterns, while the angles matched perfectly. That effect was presumably ascribed to the scrap’s partially textured structure (preferred crystal orientation along a certain crystallographic direction) which could result from mechanical machining. The microstructures of as-cast Mg–Al–Zn alloys comprise a solid solution of Al and Zn in Mg and precipitates of ternary Φ-Mg_21_(Al, Zn)_17_ and binary Mg_17_Al_12_ phases. In the latter, some of the Al atoms can be replaced by Zn and form Mg_17_(Al, Zn)_12_ [97,98]. Corrosion resistance of Mg–Al–Zn alloys is ascribed to intermetallic phase precipitates acting as barriers for grains (although, in small amounts, they may act as microgalvanic cells and enhance corrosion) and to aluminum-rich regions near the alloy surface [99,100]. So, probably, the detected Al phase could be ascribed to the existence of local Al-rich regions. For the Mg–Nd–Zn–Zr scrap component, no typical alloying elements (Nd, Zn, Zr) were identified by the XRD analysis. However, their presence was confirmed by the EDX analysis results reported above. 

For the 1 and 2 h milled samples, another phase (Mg solid solution in Al) was detected. Peak position shift and broadening were observed as well. The said changes in the XRD patterns indicated that high-energy ball milling introduced lattice microstrains and crystalline imperfections into the microstructure which are typically favorable for corrosion [92,101,102]. The XRD patterns for the samples with the additive demonstrated the presence of newly formed phases, Mg_2_Sn and Mg_2_Pb. Those intermetallic compounds had overlapping intensity peaks except those for large scattering angles (exceeding 90 degrees). Therefore, it could be expected that indeed the eutectic ‘POS-61’ alloy melted during ball milling and both its components reacted with Mg. The existence of the said intermetallic compounds was confirmed for a number of Mg-based alloys [103,104]. For the samples with 10 wt.% alloys milled for 2 and 1 h, no Sn and Pb were detected, while for that activated for 0.5 h, residual Pb was observed. According to the findings from [105], in liquid Mg–Pb–Sn solution, the Mg_2_Sn–Pb system was thermodynamically more stable as compared to Mg_2_Pb–Sn; therefore, the latter could transform into the former. That likely was the reason why after a short activation during 0.5 h, the registered unreacted component was Pb and no Sn. The samples ball milled with smaller amounts of additive differed from those with the maximum amount in that their intensity peaks for the intermetallic phases were lower and no residual Pb was detected. 

### 3.2. Tests for Hydrogen Generation Performance

#### 3.2.1. Effect of Ball Milling Duration

**Figure 4:**
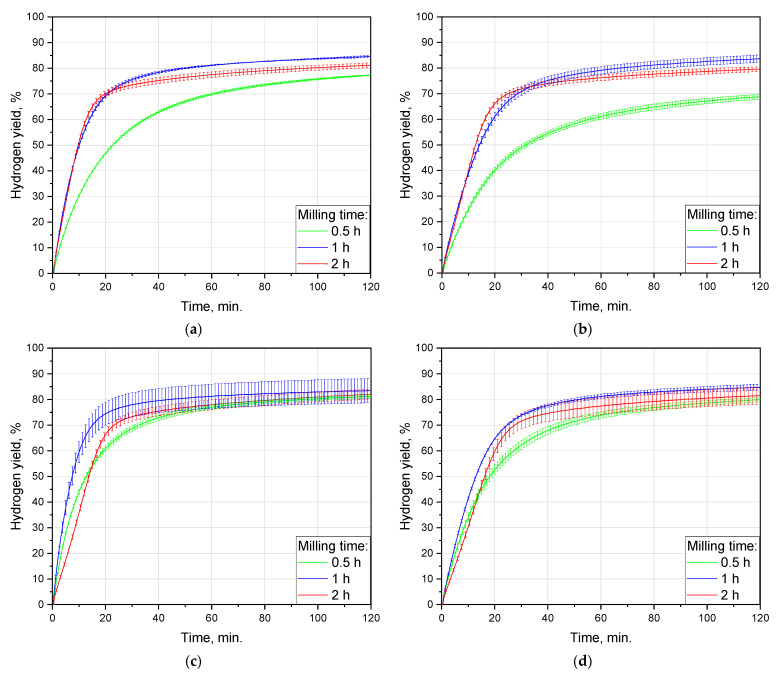
Hydrogen evolution kinetic curves for different ball milling durations and sample compositions: (**a**) no additive; (**b**) 2.5 wt.% additive; (**c**) 5 wt.% additive; (**d**) 10 wt.% additive.

The effect of ball milling duration was studied for all tested ‘POS-61’ contents: 0, 2.5, 5, and 10 wt.%. The sets of hydrogen evolution kinetic curves are shown in Figure 4, and the data on hydrogen yields and maximum reaction rates are listed in Table 2. For all of the compositions, the highest maximum hydrogen evolution rates were observed for 1 h ball milled samples: roundly 86, 63, 108, and 60 mL/min./g for 0, 2.5, 5, and 10 wt.% of ‘POS-61’, respectively. The respective values were 77, 58, 52, and 43 mL/min./g for 2 h milled samples and 56, 45, 83, and 56 mL/min./g for 0.5 h of activation. After 2 h of the experiment, the achieved hydrogen yields for the samples fell within the range of ~70–90%. The highest scrap-to-hydrogen conversion values also majorly corresponded to 1 h ball milling time and were equal to (84.6 ± 0.4)%, (83.7 ± 1.5)%, (83.5 ± 4.7)%, and (84.8 ± 1.2)% for 0, 2.5, 5, and 10 wt.% of the additive content. Activation during 2 h resulted in the corresponding figures achieving (81.2 ± 0.9)%, (79.6 ± 0.9)%, (82.0 ± 1.9)%, and (81.6 ± 3.3)%, and 0.5 h of milling provided as much as (77.3 ± 0.2)%, (68.9 ± 1.0)%, (81.2 ± 0.7)%, and (79.9 ± 0.8)%, respectively. As can be seen, the maximum hydrogen production rates for the samples activated for 2 h with 5 and 10 wt.% ‘POS-1’ alloy were the lowest as compared to those for 0.5 and 1 h of activation, while those for the samples containing 0 and 2.5 wt.% of the additive were relatively close to the respective maximums for 1 h milling. Therefore, for the tested conditions, materials, and ball milling parameters, 1 h appeared to be the optimal time. The existence of the optimal ball milling durations was reported in a number of previous studies for Mg and Al powders [89,92,94]. 

For all of the compositions, the 2 h kinetic curves had a distinctive acceleration section in the beginning (intervals from 0 to 10–15 min.), in contrast to those for 0.5 and 1 h which demonstrated only gradual deceleration over time. The possible reason for such a difference could be attributed to the partial surficial oxidation of the samples with residual oxygen during prolonged high-energy ball milling and to that MgO hydration with the formation of a less dense Mg(OH)2 took some time [106]. In studies [107,108], intensified oxidation of Mg and Al powders with metal additives (Ni, Co, Bi) by air at elevated temperatures was proven by thermogravimetric analysis. The mentioned results supported that oxidation was apparently augmented in the presence of higher activator amounts—5 and 10 wt.%—which provided the slowest maximum hydrogen evolution rates after 2 h ball milling as compared with those for all other tested activation intervals and additive contents. 

The lowest hydrogen generation rates for the 0.5 h milled samples with 0 and 2.5 wt.% ‘POS-61’ in comparison with the longer milling durations for the same compositions, presumably, resulted from a much smaller number of accumulated crystal lattice imperfections which were known to represent sites for localized corrosion attacks, e.g., pitting corrosion by the enrichment in chloride [102,109,110]. 

#### 3.2.2. Effect of Additive Content

**Figure 5:**
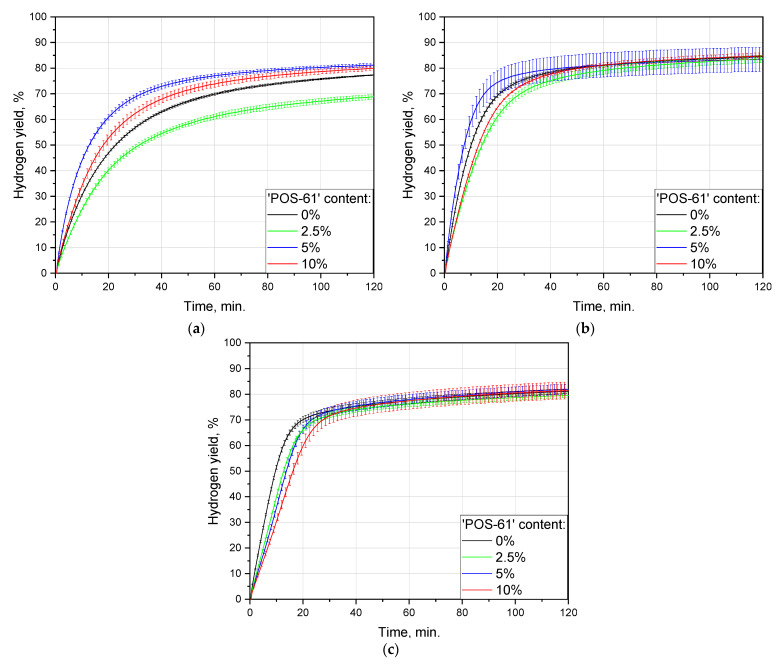
Hydrogen evolution kinetic curves for different sample compositions and ball milling durations: (**a**) 0.5 h; (**b**) 1 h; (**c**) 2 h.

The effect of the additive content for different ball milling durations is illustrated in Figure 5. For the 2 h milled samples, the gradual decrease in the maximum hydrogen reaction rate with a gradual increase in the additive content was explained in the previous subsection. As can be seen from the respective plot, for all tested additive contents the difference in their measured hydrogen yields was well within the statistical errors, as did the variance in the final hydrogen yields obtained in the case of 1 h of the activation. And for that duration, the highest hydrogen production rate corresponded to the sample with 5 wt.% of ‘POS-61’. That value for the composite containing 10 wt.% of the additive appeared to be lower than that for the powder of scrap only, and one for the 2.5 wt.% occurred to be the minimum one. For 0.5 h of milling time, the first- and second-highest maximum hydrogen evolution rates were provided, respectively, by the samples with 5 and 10 wt.% ‘POS-61’, and again the lowest value was observed for 2.5 wt.%. The same trends were for the relevant hydrogen yields.

The corrosion rate of Mg–1Sn alloy was reported to be more rapid than that for pure Mg, and the addition of 1–3 wt.% of Sn increased the corrosion and hydrogen release rate of Mg–6Al–1Zn, Mg–6Al–5Pb–0.5Mn–0.5RE, and Mg–7Al–0.2Mn alloys [111,112,113,114,115]. Contradictorily, an extruded Mg–Sn alloy with a high Sn of 5 wt% displayed improved corrosion resistance in comparison to pure Mg, and 3 wt.% of Sn provided better corrosion protection to Mg–2Al–6Zn and Mg–Zn alloys [116,117,118], and in the study [119] for Mg–Sn alloy 1.5 wt.%, Sn was optimal and provided the best corrosion resistance as compared to 0.5, 1, and 2 wt.%. Further, in [120] it was revealed that 5 and 10 wt.% Sn added to high pure Mg drastically improved its corrosion resistance. According to the results from [121,122,123,124], alloys Mg–6Al and Mg–6Al–1In modified with 5 wt.% Pb demonstrated more significant anodic reaction activity. As much as 0.1 wt.% Pb had a negative effect on the corrosion resistance AZ91 [125], its content of 0.2–1 wt.% appeared to improve that for the Mg–10Al–12Si system [126]. In [103] among the tested 0.6, 1.2, and 1.8 wt.% Pb amounts added to Mg97–Zn1–Y2 alloy the minimum one inhibited corrosion better than the larger ones, and 1.8 wt.% had almost the same self-corrosion potential as the original compound. Large amounts of Pb (~30 wt.%) were proven to inhibit the corrosion of Mg–9.2Al–0.8B alloys [127]. In the range from 2.5 to 7.5 wt.% Pb, the increase in its content improved corrosion protection for Mg–3Al, but reduced it for Mg–6Al and Mg–9Al systems [128]. The research [129] also established that low (0.1–0.5 wt.%) amounts of Pb or Sn introduced into a pure (99.95%) Mg alloy augmented its corrosion protection. So, for certain compositions, depending on their content, Pb and Sn could either enhance corrosion or improve metal protection against it.

Taking into account the listed results, the following explanation for the additive content influence for 1 and 0.5 h milled samples can be proposed. In the case of the 10 wt.% content (nearly 6 wt.% of Sn and 4 wt.% Pb), the possible explanation for the observed trends could be that, in the case of 1 h milling, such amount of the additive led to the more extensive formation of the intermetallic phases Mg_2_Sn and Mg_2_Pb. Although the said compounds were expected to act as cathodic phases and accelerate Mg corrosion in the conductive NaCl media, due to their abundance they could provide the opposite effect by the formation of precipitates along the grain boundaries [130,131,132], thus preventing the oxidation of the ‘core metal’. However, 0.5 h seemed to be insufficient time for the completion of the reaction between Mg and the additive (supported by the detected residual Pb) and, probably, for the effective distribution of the alloy components over the scrap particles as well. For the said reasons, the inhibition effect of the 10 wt.% additive could be reduced. In the case of the 2.5 wt.% ‘POS-61’, the small amounts of Sn (1.5 wt.%) and Pb (1 wt.%) could fall within the ranges beneficial for corrosion inhibition, in accordance with the findings for different Mg-based alloys listed above. Conversely, higher amounts of those elements for the 5 wt.% additive, 2 wt.% Pb and 3 wt.% Sn, were reported to barely affect the self-corrosion potential and to reduce corrosion resistance (for Mg-Al alloys), respectively. Thus, for the selected materials and under the examined ball milling and experimental conditions, 5 wt.% was, presumably, the optimal additive content for 0.5 and 1 h milled samples as compared to 0, 2.5, and 10 wt.%.

### 3.3. Characteristics of the Reaction Products

**Figure 6:**
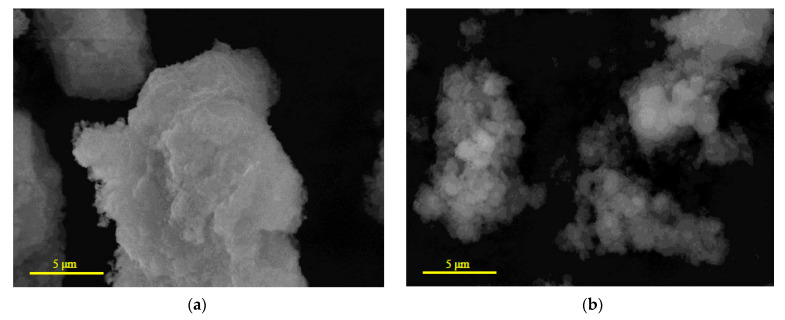
Microstructure of the solid reaction product obtained from different samples: (**a**) no additives; (**b**) 5 wt.% ‘POS-61’.

**Figure 7:**
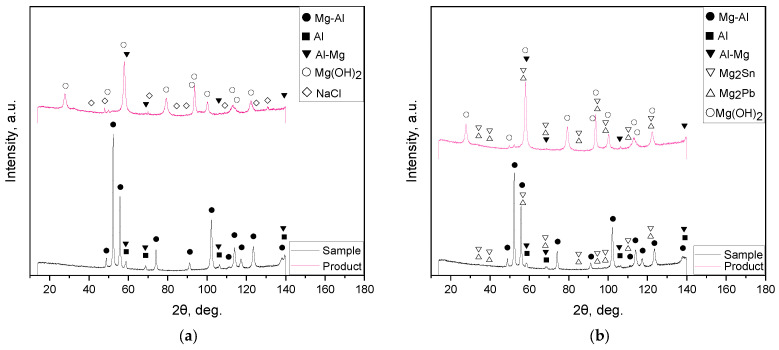
XRD patterns of the solid reaction product obtained from different samples: (**a**) no additives; (**b**) 5 wt.% ‘POS-61’.

The solid reaction products were investigated for the 1 h ball milled samples without additives and with 5 wt.% ‘POS-61’. The samples’ particles covered with the reaction product are shown in Figure 6. As can be seen, the solid product formed clusters of small flake-like structural elements. The XRD patterns for the same reacted samples are represented in Figure 7. As expected, the product majorly comprised Mg(OH)_2_. Some amount of non-reacted Al-Mg solid solution was identified as well. In the product obtained from the sample without additive, residual NaCl from the solutions was detected, while in that activated with 5 wt.% additive its peaks were not definitely observed (probably, it was better rinsed with water). The latter also contained the intermetallic phases, Mg_2_Sn and Mg_2_Pb.

## 4. Conclusions

Twelve sorts of hydroreactive samples were manufactured of the mixed Mg-based scrap with 0, 2.5, 5, and 10 wt.% low melting point Sn–Pb soldering alloy by high-energy ball milling during 0.5, 1, and 2 h. Their hydrogen production performances were tested in 3.5 wt.% aqueous NaCl solution at room temperature. The effects of milling time and additive content were investigated.

The major effects of ball milling were the introduction of imperfections to the metal crystal lattice increasing the material’s vulnerability to pitting corrosion, microstructural transformation of the particles from flat shapes to equiaxial solid objects with a decrease in the specific surface area, and, presumably, partial surficial oxidation of the particles with residual oxygen resulting in lower reaction rates in the beginning of the process. On the one hand, prolonged activation (2 h) created more lattice imperfections. But, on the other hand, it promoted oxidation, especially for the samples containing 5 and 10 wt.% additive, and ‘reshaped’ the original flakes with extended surfaces into compacted agglomerates of smaller particles generated by their fracturing and cold welded to each other. The shorter milling intervals (0.5 and 1 h), most likely, provided less accumulation of the strain energy in the crystal lattice, but did not result in notable oxidation or reduction of the specific surface area. For all samples, 1 h occurred to the optimal activation time corresponding to the maximum hydrogen yields and reaction rates.

The influence of the additive content appeared to be nonmonotonic. Among all samples ball milled for 0.5 and 1 h, the lowest hydrogen generation rates were obtained for 2.5 wt.% ‘POS-61’. The highest ones corresponded to 5 wt.% of the additive, and the second- and third-highest were observed, respectively, for the 0.5 and 1 h milled samples with the highest content of 10 wt.%. Such results were potentially caused by a complex effect of Sn and Pb on the corrosion resistance of Mg-based alloys. Although the results of the preceding studies on that subject were somewhat contradictory, it was generally reported that large amounts of Sn and Pb could increase the corrosion protection of alloys due to the formation of extended intermetallic—Mg_2_Sn and Mg_2_Pb—precipitates along grain boundaries. Low amounts of additives could improve corrosion stability as well. However, within some content ranges (e.g., 2–3 wt.% of Sn or Pb), the formed intermetallic phases could enhance corrosion of the basic metal due to the formation of microgalvanic cells between the intermetallic and Mg phases without ‘shielding’ the grains from the oxidizing solution. In the present study, 5 wt.% of Sn–Pb was found to be optimal.

The results of the study demonstrated that the Mg scrap-based hydroreactive sample with the highest hydrogen evolution rate and yield corresponded to the combination of the optimal milling time of 1 h with optimal additive content of 5 wt.%. In situ hydrogen generation by the oxidation of mixed Mg-based scrap with seawater (herein, simulated with 3.5 wt.% NaCl solution) can be a beneficial method for the valorization of such a complex sort of waste. Taking into account the corrosion-resistant behaviors of the tested Mg–Nd–Zr–Zn and Mg–Al–Zn alloys, their relatively high contents of alloying elements, and small impurity fractions, the proposed approach to their utilization may appear to be rather reasonable.

## Figures and Tables

**Table 1:** EDX spectra for the selected analyzed regions.

Sample	Spectra No.	Mg	Al	Zn	Nd	Zr	Pb	Sn	Fe	Cr	Mo	Si	Cu	Sb
10 wt.% ‘POS-61’, 2 h	2	35.2 ± 0.3	-	-	0.9 ± 0.2	-	25.4 ± 0.4	38.5 ± 0.3	-	-	-	-	-	-
	4	11.8 ± 0.2	-	-	-	-	-	-	62.9 ± 0.3	24.6 ± 0.2	0.8 ± 0.2	-	-	-
10 wt.% ‘POS-61’, 1 h	17	65.6 ± 0.4	-	0.6 ± 0.2	3.0 ± 0.2	30.8 ± 0.4	-	-	-	-	-	-	-	-
	21	74.4 ± 0.3	17.9 ± 0.2	-	2.6 ± 0.2	-	-	0.9 ± 0.1	-	-	-	4.2 ± 0.1	-	-
10 wt.% ‘POS-61’, 0.5 h	28	66.4 ± 0.3	4.0 ± 0.1	2.0 ± 0.2	27.5 ± 0.3	-	-	-	-	-	-	-	-	-
	30	60.6 ± 0.4	3.3 ± 0.1	0.6 ± 0.2	3.2 ± 0.2	31.8 ± 0.4	-	-	0.5 ± 0.1	-	-	-	-	-
5 wt.% ‘POS-61′, 2 h	36	47.9 ± 0.3	-	-	1.8 ± 0.2	-	17.3 ± 0.4	32.1 ± 0.3	-	-	-	-	0.9 ± 0.2	-
5 wt.% ‘POS-61′, 1 h	46	15.1 ± 0.2	23.0 ± 0.2	-	-	-	14.1 ± 0.4	46.6 ± 0.3	-	-	-	-	-	1.2 ± 0.3

**Table 2:** Summarized experimental data for the samples with various compositions and ball milling durations.

Composition	Ball Milling Time, h	Hydrogen Yield, %	Maximum Reaction Rate, mL/g/min.
Scrap without additives	0.5	77.3 ± 0.2	56
	1	84.6 ± 0.4	86
	2	81.2 ± 0.9	77
Scrap + 2.5 wt.% ‘POS-61’	0.5	68.9 ± 1.0	45
	1	83.7 ± 1.5	63
	2	79.6 ± 0.9	58
Scrap + 5 wt.% ‘POS-61’	0.5	81.2 ± 0.7	83
	1	83.5 ± 4.7	108
	2	82.0 ± 1.9	52
Scrap + 10 wt.% ‘POS-61’	0.5	79.9 ± 0.8	56
	1	84.8 ± 1.2	60
	2	81.6 ± 3.3	43

## Data Availability

Not applicable.

## Appendix A

EDX analysis results for the selected spots on the samples ball milled with 10 wt.% ‘POS-61’ during 2, 1, and 0.5 h are shown in Figure A1.
